# Cost-effectiveness analysis of the available strategies for diagnosing malaria in outpatient clinics in Zambia

**DOI:** 10.1186/1478-7547-7-5

**Published:** 2009-04-08

**Authors:** Pascalina Chanda, Marianela Castillo-Riquelme, Felix Masiye

**Affiliations:** 1 National Malaria Control Centre, Box 32509, Lusaka, Zambia; 2 Health Economics Unit, Department of Public Health and Family Medicine, University of Cape Town, Cape Town, South Africa; 3 Department of Economics, University of Zambia, Lusaka, Zambia

## Abstract

**Background:**

Malaria in Zambia accounts for about 4 million clinical cases and 8 000 deaths annually. Artemether-lumefantrine (ACT), a relatively expensive drug, is being used as first line treatment of uncomplicated malaria. However, diagnostic capacity in Zambia is low, leading to potentially avoidable wastage of drugs due to unnecessary anti malarial treatment.

**Methods:**

A cost-effectiveness evaluation of the three current alternatives to malaria diagnosis (clinical, microscopy and Rapid Diagnostic Tests- RDT) was conducted in 12 facilities from 4 districts in Zambia. The analysis was conducted along an observational study, thus reflecting practice in health facilities under routine conditions. Average and incremental cost effectiveness ratios were estimated from the providers' perspective. Effectiveness was measured in relation to malaria cases correctly diagnosed by each strategy.

**Results:**

Average cost-effectiveness ratios show that RDTs were more efficient (US$ 6.5) than either microscopy (US$ 11.9) or clinical diagnosis (US$ 17.1) for malaria case correctly diagnosed. In relation to clinical diagnoses the incremental cost per case correctly diagnosed and treated was US$ 2.6 and US$ 9.6 for RDT and microscopy respectively. RDTs would be much cheaper to scale up than microscopy. The findings were robust to changes in assumptions and various parameters.

**Conclusion:**

RDTs were the most cost effective method at correctly diagnosing malaria in primary health facilities in Zambia when compared to clinical and microscopy strategies. However, the treatment prescription practices of the health workers can impact on the potential that a diagnostic test has to lead to savings on antimalarials. The results of this study will serve to inform policy makers on which alternatives will be most efficient in reducing malaria misdiagnosis by taking into account both the costs and effects of each strategy.

## Background

Malaria is a major public health problem in the world where at least 3.2 billion people are at risk of the disease annually [1]. The World Health Organisation (WHO) estimates that 60% of the cases and 80% of malaria related mortality occurs in Sub Sahara Africa (SSA) [2] an area geographically defined as the hub of poverty.

In Zambia, the disease is endemic countrywide and about 95% of all cases are caused by the mostly deadly malaria parasite species, *Plasmodium falciparum*[3]. The Health Management Information System (HMIS) estimates 4 million clinical cases and 8,000 deaths due to malaria annually. It is against this background that in 2003, the national antimalarial drug policy in Zambia was revised. This led to the replacement of the failing chloroquine (CQ) and Sulphadoxine-pyrimethamine (SP) with artemisinin-based combination therapy (ACTs) for the treatment of uncomplicated malaria. Currently, ACTs have been scaled up countrywide to treat uncomplicated cases of malaria. ACTs have been reported to be highly efficacious in treating uncomplicated malaria and consequently reducing the transmission of resistant genes [4,5].

Nonetheless, malaria diagnostic capacity plays a pivotal role in correctly identifying malaria cases from non-malaria cases. The use of an accurate diagnostic test, which is determined by its sensitivity and specificity, would imply that only true cases would be prescribed an antimalarial. This would help in channelling antimalarial drugs to those that need them and at the same time provide the non-malaria cases an opportunity to be examined for other causes of illness. However, this is a challenge for Zambia where only 34% of the facilities have laboratory facilities for microscopy services and of these only 60% have functional laboratories [6]. Thus, most fevers are being diagnosed clinically to be malaria. Integrated management of childhood illnesses (IMCI) guidelines are being applied to ensure that other causes of fever in children are excluded [7,8]. However, these guidelines have been found to be misapplied, possibly because only 33% of the frontline health workers have received IMCI training [9].

Coartem^® ^(a fixed dose combination of Artemether- lumefantrine -AL), which is being used to treat uncomplicated malaria in Zambia, is much more expensive than the former monotherapies. Thus, the malaria drug budget in Zambia has increased almost eight-fold from US$ 579, 300 in 2003 (when SP was the first line treatment) to US$ 4,474,018 in 2005 (when AL was scaled up country wide). Without malaria confirmation, it is difficult to exclude fevers, which are not due to malaria, thus the true burden of the disease proves difficult to quantify. This might be lead to wastage of drugs on unnecessary treatment and inappropriate patient management.

New technologies on malaria diagnosis have introduced Rapid Diagnostic Tests (RDTs), which work on the principle of antigen detection methods. These immunochromatographic dipsticks can be sensitive to two basic antigens of the malaria parasites; the histidine-rich protein-2 (HRPII) or parasite lactate dehydrogenase (pLDH) [10]. These tests are now being thought of as a viable option for defining malaria parasite presence in the patients suspected of having malaria. RDTs, unlike microscopy, can easily be used by any frontline health workers and do not need extra infrastructure [11].

In this context, it is relevant to assess the diagnostic accuracy (intermediate outcome) with the economic implications of the available diagnostic techniques for malaria. On the basis of cost effectiveness, the study seeks to challenge the current reliance on clinical diagnosis as opposed to the introduction of malaria confirmatory diagnostic methods in this era of ACTs.

## Methods

### Study design

This study evaluates the operational cost-effectiveness of the three available options (clinical, microscopy and RDTs) for diagnosis of malaria in light of ACTs as first line treatment. This study was conducted from a public health (or provider) perspective mainly because malaria services in Zambia are provided free of charge (with the exception of registration costs in urban centres). It was also assumed that since each district implemented all the three strategies, the indirect costs borne by patients would be similar across diagnostic strategies. The study was conducted in the context of the routine health facility operations as per standard malaria treatment guidelines in Zambia. The outcome measure, *the proportion of cases correctly diagnosed *is an intermediate one. It includes cases found positive in the presence of the condition and cases found negative in the absence of the condition in relation to the total cases diagnosed by each method.

### Study population and period of evaluation

All malaria related visits (suspected or confirmed), which occurred from March to November 2005 in the selected 12 facilities were included in the study. This timeline allowed for the capture of both the low and high transmission seasons of malaria in Zambia. The method of diagnosis of each patient depended on the predetermined diagnosis strategy (clinical, microscopy or RDT) assigned to the facility prior to the commencement of the study. However, the management of the patient and the type of treatment administered was left to the health workers' decision. In other words, in the case of laboratory or RDT confirmation, the study team did not indicate a strict treatment prescription rule based on the test result. Efforts were made to ensure that all the health staffs at each facility were trained in data recording and use of RDTs (where applicable) based on standard job aids and the national malaria case management guidelines.

### Study sites

In the four district selected for the study; Chingola (Copperbelt Province), Kabwe (Central Province), Kalomo (Southern Province) and Chongwe (Lusaka Province) malaria is meso to hyper endemic. Three facilities in each selected district were assigned one of each malaria diagnostic approaches: clinical, microscopy or RDTs, bringing the total number of facilities studied to 12. These sites were also part of the sentinel sites surveillance system for malaria; this ensures that the different epidemiological zones in Zambia were represented. Likewise, the districts selected were part of larger study collecting information on the "financial sustainability plan (FSP)" for scaling up malaria control activities. This opportunity provided the means of collecting quality and reliable data from these facility registers under routine conditions.

### Description of the interventions under comparison

#### Clinical Diagnosis of Malaria

This strategy is carried out for a trained health worker who can diagnose malaria based on the signs and symptoms a patient presents with. The minimal elements required for clinical diagnosis are simply a thermometer (for measurement of axillary temperature) and a weighing scale where applicable. If temperature is above or equal to 37.5°C or where a history of fever exists and malaria is suspected, treatment is commenced and the patient returns home. Thus it is possible for a trained health worker to exclude fevers from malaria based on the patients' signs and symptoms. Cases clinically thought not to have a fever due to malaria are considered 'negative'.

#### Microscopy Diagnosis of Malaria

Where microscopy facilities are available, a clinical officer or nurse initially assesses patients. If malaria is suspected, the patient is sent to the laboratory for malaria investigation. A laboratory technician or microscopists analyses the patients' blood sample for malaria infection. The results are recorded in the patients file and the patient is instructed to return to the screening room with the laboratory results. The clinician then prescribes treatment based on both the laboratory result and the clinical presentation of the patient at that time. This strategy required optimal laboratory infrastructure, including a trained microscopist or laboratory technician, a functional microscope, reagents, electricity supply, water supply and other consumables such as lancets, blood slides. Microscopy diagnosis results are obtained after at least 30 minutes.

#### RDT Diagnosis of Malaria

A clinical officer or nurse initially assesses the patient, once malaria is suspected; parasitological confirmation of malaria infection is performed with an RDT. Depending on the results, the clinician may then prescribe an antimalarial. It should be noted here that the health worker performs both the clinical assessment and the RDT test. This is unlike microscopy facilities where laboratory personnel are essential in the diagnosis of malaria. The minimum requirements for this diagnostic strategy include: 1 RDT kit (which contains a test dip stick, desiccant, sample applicator, buffer solution and collection capillary tubes) and a clinical officer or nurse (or Commissioned Daily Employee in some rural areas) on how to use the RDTs. Lancets, methylated spirit and cotton wool are some of the supplies needed to be bought separately if they do not come with the kit.

### Data collection procedures

During the study period, all the patients suspected of malaria were being recorded in the facility's outpatient malaria registers and received clinical or confirmatory diagnosis based on the allocated method in that facility. In all the sites AL was being used as first line treatment for malaria. Records were kept for all the patients screened on the diagnostic strategy (clinical, microscopy or RDT), test result (positive or negative), malaria type (uncomplicated or complicated), antimalarial treatment given (quinine, SP or AL) and referrals. The facility registers thus provided a basis for morbidity data collection (malaria suspected outpatient visits and confirmed malaria cases). Secondary data from published literature was used to determine the sensitivity of clinical, microscopy and RDTs in diagnosing malaria.

In the selected health facilities, three levels of supervision were put in place to ensure data completeness and accuracy. The first level corresponded to the health facility head to supervise daily and weekly patient data profiles, district heath information officers in turn (second level) supervised facility heads and conducted on spot check of patient files and ensured they were consistent with malaria registers. Finally, the central level teams supervised monthly data collection on site and on data entry files.

Cost information was obtained from facilities, central level sources and suppliers of commodities where applicable. The ingredient approach combined with step-down approach to costing was used to estimate average costs per month and per year [12]. Data collection forms were developed to conduct inventories on capital and recurrent costs related to malaria diagnosis. Health staffs were also interviewed to get an opinion on some of the resources required the daily management of malaria patients. Financial reports, cash receipts, malaria outpatient registers, district action plans, procurement units, market prices of commodities and various data sources were reviewed and triangulated to accurately measure and value the resources used. Cost data, was obtained from various expenditure points such as obtained from district or central level sources, expenditure reports and market prices of goods. The cost of distribution was estimated from main government distributors and was added to the unit cost.

#### Capital costs

Capital resources (i.e. items which have a useful life of more than one year) were annualised based on the replacement value, its estimated useful life and the official discount rate (5%) used in Zambia (MOH Planning Unit, Zambia personal communication). Capital costs comprise equipment, vehicles and buildings. The allocation of capital costs to malaria diagnosis was determined by estimating an allocation rate per facility. This was derived from malaria OPD utilisation in relation to all the visits registered in the facility. However, laboratory related capital costs were allocated based on the number of analyses for malaria as a proportion of the total laboratory analyses for all diseases.

#### Recurrent costs

Personnel costs were measured based on number and categories of each type of staff (nurse, clinical officer, medical doctor, community health worker, etc) and their respective annual salaries. These were then allocated based the utilisation of facilities by suspected malaria patients.

Shared recurrent costs such as supplies, and utilities were valued using a step-down approach to costing and allocated based on the facility utilisation by malaria patients. However, costs unique to malaria (such as cost of the diagnostic technique) were fully allocated as such. Districts are also allowed up to 15% of their total expenditures on administration costs (or overheads). Therefore in the absence of a better sources of administrative expenditures, it was assumed that on average, 15% of malaria related expenditure would be on administrative costs such as fuel, communications, cleaning materials, stationery and other utilities. For simplicity, all other recurrent costs (non-personnel or malaria specific) were termed overheads in this study. Table 1 summarises the various assumptions and parameters used in the analysis of costs and cases correctly diagnosed.

**Table 1:** Parameter assumptions and data sources

**Description**	**Assumption/Parameter**	**Source**
Exchange rate	1 USD = ZMK4512.51 (All costs are presented in US$)	(average March to November 2005)

Discount rate	5%	MOH Planning Unit

Overhead costs	15% (Of district recurrent expenditure)	District Health Office (DHO)

Personnel costs	Gross earnings (Collected from central level, allocated based on malaria utilisation)	MOH/DHO

**Cost of drugs and tests**		

AL	2.45 USD	NMCC, (weighted average cost per person/course including storage and distribution costs).
SP	0.18 USD	
Quinine	0.84 USD	

RDT	1.50 USD	NMCC (excludes personnel and capital costs).
Microscopy	1.00 USD	

Laboratory utilisation	60%	Expert opinion

**Sensitivity of the diagnostic techniques**		

Clinical	100%, 90%	Current study data from clinical sites and published literature [13,14].

Microscopy	91%	Colin et al 2002 [15]

RDTs	95.4%	Guthman et al 2002 [16] Mendiratta et al 2006 [17].

Malaria prevalence by district*		

Chingola	18.8%	NMCC 2005 [9]

Chongwe	22.0%	NMCC 2005 [9]

Kalomo	26.3%	NMCC 2004

Kabwe	10.6%	NMCC 2005 [9]

The malaria prevalence above refers to the proportion of people with detectable malaria parasites in their peripheral blood, approximated from the average annual parasite prevalence surveys among the 2–9 years old in each district.

#### Outcome measures

Malaria diagnosis accuracy of each technique was evaluated by its ability to increase cases correctly diagnosed (true positives and true negatives) and the ability to decrease cases incorrectly diagnosed (false positives and false negatives). These were calculated from the total number of patients screened, the screening results, the underlying malaria prevalence and the sensitivity of the diagnostic strategy used. A '2 x 2 Table' (which is based on Bayesian theory applied on screening methodology) was used to carry out these calculations.

The main outcome measure was the number and proportion of malaria cases correctly diagnosed by each diagnostic strategy. The sensitivity of each strategy was drawn from evidence from the literature and weighted up according to sample size and relevance for the Zambian setting. Thus, sensitivity was used as the input parameter, whereas specificity was an output variable. This is because sensitivity and specificity vary with prevalence, and the districts under study had varying underlying prevalence as shown in table 2. For clinical diagnosis, the sensitivity for two sites (Kalilo and Kalonda) was assumed at 100%, because almost all the suspected malaria visits were classified as positive for malaria. For the remaining two clinical sites (Chinyunyu and Natuseko), which at least reported on some negative cases, the average sensitivity was assumed at about 90%. These figures were similar to sensitivity analysis from literature [13,14].

**Table 2:** Facility visits and diagnostic results

District	Health Facility	Total Visits	Diagnostic Result			% found Negative of total visits	Diagnostic Method
					
			Positive Uncomplicated	Positive Severe	Negative		
Chingola (Urban)	Kalilo	409	378	29	2	0.49	Clinical
	
	Kabundi	3084	1552	15	1517	49.19	Microscopy
	
	Kasompe	1187	281	6	900	75.82	RDT

Chongwe (Rural)	Chinyunyu	1430	1367	11	52	3.64	Clinical
	
	Chongwe	3338	1130	46	2162	64.77	Microscopy
	
	Chalimabana	2634	928	35	1671	63.44	RDT

Kalomo (Rural)	Kalonda	1020	1018	2	0	0.00	Clinical
	
	Namwianga	1975	813	0	1162	58.84	Microscopy
	
	Mukwela	874	263	1	610	69.79	RDT

Kabwe (Urban)	Natuseko	3661	3003	21	637	17.4	Clinical
	
	Makululu	2063	282	0	1781	86.33	Microscopy
	
	Kawama	1990	216	1	1773	89.1	RDT

TOTAL		23665	11231	167	12267	51.84	-

Microscopy is assumed to be the gold standard only under ideal conditions. However, under routine conditions, microscopy has been found to have sensitivity of 91% and specificity of 71% [15] when compared to expert microscopy. Thus, the sensitivity rate from these findings was used to determine cases correctly diagnosed through microscopy.

For RDT tests, the weighted average of the sensitivity was calculated from studies that used Paracheck Pf brand and performed field evaluations by comparing RDT to expert microscopy [16,17]. The sample size of each study determined the weight used in the calculation of the average sensitivity. The two studies were selected based on clinical and methodological similarities. In this way, it was hoped that statistical heterogeneity would be reduced. The weighted average sensitivity for RDT was 95.36%.

The underlying prevalence in the districts facilities was obtained from survey data conducted by the NMCC. These prevalence values are assumed to approximate the true annual prevalence of malaria among patients suspected of malaria seeking care at the facility. An important aspect of these surveys is that they incorporate the 2–9 years who are the standard group for estimating malaria parasite prevalence [9]. In the case of Chongwe, Kabwe and Chingola, the prevalence figures were obtained from the 2005 parasitological surveys, whereas for Kalomo, the 2004 figure was used in the absence of any latest estimates (see table 1).

Thus based on the sensitivity of each strategy and the underlying prevalence in each district, the following equations (derived from Bayesian theory) were used to estimate true positives, false positives, true negatives and false negatives and consequently the cases correctly diagnosed.

1. True positives = prior prevalence * visits * sensitivity

2. False positives = found positive - true positives

3. False negatives = (prior prevalence * total visits) - true positives

4. True negatives = found negative - false negatives

5. Cases correctly diagnosed = true positives + true negatives

6. Accuracy = number of cases correctly diagnosed/total visits.

##### Average Cost Effectiveness and Incremental Cost Effectiveness Analysis

After establishing the costs and consequences of each alternative, the average cost per case diagnosed as well as the average cost per case correctly diagnosed was calculated for each strategy. Average costs were calculated with and without treatment costs. However, the relevant cost effectiveness ratio has been defined as the average cost per case correctly diagnosed and treated, as follows:



Where,

C_d _= Cost of diagnosis

C_t _= Cost of all treatment

CCD = Number of cases correctly diagnosed.

The incremental cost per additional case correctly diagnosed was calculated based on the changes in the costs and effects of moving from the strategy that costs less per patient diagnosed to the next alternative in order of the rank of costs per patient. Thus:



##### Sensitivity analysis

Simple (one-way) sensitivity analysis was used on parameters that as demonstrated elsewhere [12,18,19] might impact on the study results. These include; discount rate, the sensitivity of clinical diagnosis, accuracy of diagnostic tests, personnel costs, allocation factor for shared costs and prices of RDTs and AL. Personnel costs were chosen because they were a major cost component in all the facilities. When performing sensitivity analysis, ACER values were recalculated maintaining the observed drug prescription practices.

### Data entry and analysis

Morbidity data was entered and analysed in STATA version 8. Cost data was entered and analysed in excel following the principles of cost analysis [12,19]. The cost of malaria drugs for treatment was estimated from the unit cost of antimalarials and the number of patients treated by each type of antimalarial. The potential costs of scaling up the most cost effective strategy in the entire district of analysis were based on the already existing structures and resources.

## Results

### Summaries from morbidity data

During the study period, (March to November 2005), more than 23,600 suspected malaria visits were recorded at the 12 out-patient clinics in the four districts. Of these attendances, 6520 (28%) were reported at clinical facilities, 10460 (44%) at microscopy facilities and 6685 (28%) at the RDTs facilities. Table 3 shows the aggregated diagnostic results for the entire study period per facility. Variations on total visits across facilities are explained partly by different catchment areas and levels of utilisation. Children under five years accounted for 51% of all attendances.

**Table 3:** Summary of average effectiveness of each strategy

Strategy	Clinical	Microscopy	RDT
Total Visits	6520	10460	6685

**Test Results**			

Found Positive (%)	5829	3838	1731

Found Negative (%)	691	6622	4954

**Estimations of Accuracy (refer to methods)**			

True Positives	977	1866	1186

False Positives	4852	1972	545

True Negative	621	6237	4896

False Negative	70	186	58

Sensitivity (%)- input	90	90.9	95.3

Specificity (%)- output	11.3	76.5	90

Cases correctly diagnosed	1598	8303	6082

Accuracy (%)	24.5	79.4	91.0

**Estimations of Reliability**			

Likelihood ratio positive	1.1	3.9	9.5

Likelihood ratio negative	0.6	0.1	0.1

Positive post-test probability	17%	53%	70%

Negative post-test probability	10%	3%	2%

Overall, regardless of diagnostic strategy, 51.84% (N = 12,267) were found not to have malaria. Another, 48.2% (N = 11398) were found to have malaria. Of those found with malaria, 98.5% were considered to be uncomplicated malaria while 1.5% (N = 167) were diagnosed with severe malaria.

### Effectiveness Analysis: Cases Correctly Diagnosed (CCD)

Table 4 summarises the estimation of CCD aggregated by each diagnostic technique.

**Table 4:** Cost profiles per diagnostic strategy

	Clinical	Microscopy	RDT
a. Catchment	28528	82434	35260

b. Visits	6520	10460	6685

Utilisation rate (b/a)	0.23	0.16	0.19

**Costs (US$)**			

Diagnostic Technique	0	11,611	10,302

Personnel	14,899	55,347	15,828

Personnel (extra time)	0	0	490

Capital (Routine – not unique to malaria)	678	7,136	625

Capital (Laboratory- diagnosis specific)	0	1,054	-

Overheads	2,287	10,956	4,293

**Total Cost**	**17,864**	**86,103**	**31,508**

**Unit cost per type of resource**			

Diagnostic Technique	2.3	1.1	1.5

Personnel	0.1	5.3	2.4

Personnel (extra time)	0	0	0.1

Capital (Routine)	0	0.7	0.1

Capital (Laboratory*)	0	0.1	-

Overheads	0.4	1	0.6

Cost per visit	2.7	8.2	4.7

Clinical diagnosis of malaria was found to have the lowest accuracy (24%) in diagnosing malaria when compared to either microscopy or RDT methods, table 4 refers. The proportion of false positives in clinical diagnosis was more than those by microscopy and RDT strategy. The RDT diagnosis led to less false negatives (<1%), while clinical and microscopy were responsible for 1.1% and 1.8% false negatives respectively. A lower proportion of false negatives are desirable in malaria diagnosis due to the negative consequences of leaving malaria untreated.

A positive malaria case diagnosed microscopically had a 53% certainty that it was a true positive malaria case, while a negative result was 3% likely to be a true malaria case (false negative). Thus a negative malaria result diagnosed by microscopy would be more reliable than a positive result. The average accuracy of microscopy in diagnosing malaria patients was found to be about 79% (see 4 below). On the other hand, a positive malaria result on RDT had a 70% chance of being a true malaria case, while a negative result had a 2% likelihood of being a true malaria case. Both the positive and negative likelihood ratios of the RDT indicate that a malaria test result on RDT is more reliable. Among the patients diagnosed by RDT, only 8% were false positives.

### Cost Estimates by Diagnosis Strategy

The costs of malaria diagnosis were grouped into five main categories. These were personnel, capital costs, diagnostic technique and overheads. In the first step, treatment costs were not included. These categories were further specified for each diagnostic strategy and facility. All costs are expressed in USD as shown in table 4, 5, 6.

**Table 5:** Costs and cost-effectiveness ratios

	Clinical	Microscopy	RDT
a. Visits	6520	10460	6685

**Diagnosis Costs **			

b. Cost of Diagnosis (prior treatment)	17,864	86,103	31,508

***c. Cost/patient diagnosed (b/a)***	***2.7***	***8.2***	***4.7***

**Treatment Costs**			

d. Treatment costs (All treated as observed in study*)	9,422	12,708	7,918

e. Treatment costs (If only positive treated**)	9,429	5,590	3,226

**Total Costs**			

f. Total cost (b+d)*	27,286	98,811	39,426

g. Total cost (b +e)**	27,293	91,693	34,734

h. Cost/patient diagnosed and treated (f/a)*	4.2	9.4	5.9

i. Cost/patient diagnosed and treated (g/a)**	4.2	8.8	5.2

**Total Effectiveness**			

j. Number of cases correctly diagnosed	1598	8303	6082

k. Proportion of cases correctly diagnosed (j/a)	0.25	0.79	0.91

**Average Cost Effectiveness Ratios (ACER)**			

l. Total cost/cases correctly diagnosed (f/j)*	***17.1***	***11.9***	***6.5***

m. Total cost/cases correctly diagnosed (g/j)**	***17.1***	***11.0***	***5.7***

**Table 6:** Incremental cost-effectiveness ratios

Incremental Cost Effectiveness Ratios	Clinical	Microscopy	RDT
***Cost/patient diagnosed***	***2.7***	***8.2***	***4.7***

Effectiveness per case diagnosed	0.25	0.79	0.91

Moving from clinical to either microscopy or RDTs			

Incremental costs	-	5.5	2.0

Incremental effects	-	0.54	0.66

ICER		10.2	3.0

Cost/patient diagnosed and treated*	***4.2***	***9.4***	***5.9***

Effectiveness per case diagnosed	0.25	0.79	0.91

Moving from clinical to either microscopy or RDTs			

Incremental costs	-	5.2	1.7

Incremental effects	-	0.54	0.66

**ICER (USD)**		**9.6**	**2.6**

Personnel costs were an important cost component among the three strategies. While clinical and RDT personnel costs were similar at about USD 2.3 – 2.4 per visit, microscopy personnel costs were found to be the highest at USD 5.3 per visit (with considerable variation across facilities). Routine capital costs were also similar for clinical and RDT strategies but for microscopy they were 7 times higher. There was no cost associated to the diagnostic technique for clinical strategy, USD 1.2 for microscopy and higher for RDT at USD 1.6. Overheads were lowest in the clinical strategy and highest in the microscopy strategy.

**Figure 1:**
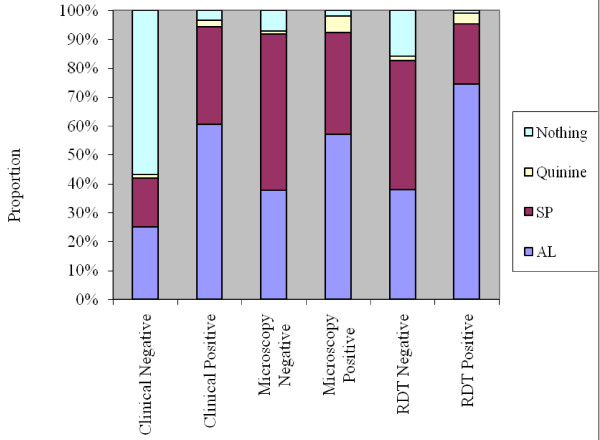
**Treatment characteristics**.

Overall, the unit cost per visit was USD 2.7, USD 8.2 and USD 4.7 for clinical, microscopy and RDT strategy respectively. A relevant finding of this study is that in general, health workers do not base the drug prescription on the test result (for both laboratory and RDTs) as shown in figure 1.

For this reason, the cost per case diagnosed (total cost of diagnosis/visits) was estimated using the total cost while excluding treatment costs at first. Then later, when estimating the cost per case correctly diagnosed, treatment costs were included in the total costs. In order to show the difference between the observed practice of treating all as observed in the study and a scenario where only cases that are found positive were treated, the results are presented using these two scenarios for treatment.

Table 5 below shows the costs and cost effectiveness ratios for the three strategies. The average cost per patient undergoing malaria diagnosis was found to be lowest in the clinical strategy (USD 2.7). The cost of microscopy was three times the cost of clinical diagnosis and twice the cost of the RDT strategy per patient diagnosed.

The potential savings on treatment if only cases found positive are treated were zero for clinical, 56% for microscopy and 59% for RDT strategy respectively. This shows that using clinical diagnosis may not lead to cost savings on treatment, while using microscopy or RDT result for treatment prescription has the potential to maximise savings on antimalarial drugs.

The ACER per case correctly diagnosed was highest in the clinical strategy, followed by microscopy and least in the RDT strategy. As expected, considering the two situations described above (treatment as observed and treatment if only found positives are treated), the ACER under the observed treatment pattern (row l) was higher than when only cases found positive are treated (row m), again except for the clinical strategy. Table 6 shows the incremental cost-effectiveness ratios for the three strategies.

Incremental analysis was estimated using as baseline the least cost-effective strategy (clinical) and moving to either microscopy or RDT. Given the differences in the number of patients seen by each strategy, cost and effect have been expressed per patient. Hence, as shown in table 6 above, the clinical strategy was used as baseline. When considering only diagnoses costs, the incremental cost required per additional case correctly diagnosed was found to be lower for RDT (USD 3) than microscopy (USD 10). In other words, microscopy should be eliminated by extended dominance [20]. When considering the incremental cost per additional case correctly diagnosed and treated, which was considered as the baseline ICER results (*), values reduced from USD 3 to USD 2.6 for RDT and from USD 10.2 to USD 9.6 for microscopy as shown in table 6. These decreases are due to the fact that, in comparison to clinical diagnosis, treatment patterns with RDTs and Microscopy generates some savings on antimalarials.

### Sensitivity Analysis

Overall, the parameters that had the strongest effect on reducing RDT efficiency against the alternatives were increases in RDT and AL costs, reducing accuracy of the RDT, an increase in the malaria allocation factor (malaria visits as a proportion of all OPD visits and increasing personnel costs. On the other hand, the parameters that improve even more the position of RDT were lower unit costs of RDT and AL and reduced malaria related visits.

## Discussion

In different epidemiological settings and variable contexts, the clinical diagnosis of malaria was not a cost effective strategy for malaria diagnosis in the four districts in Zambia, if cases correctly diagnosed were to be maximised. The RDT was found to be cheaper at correctly diagnosing malaria (USD 6.5) than microscopy (USD 11.9) and clinical (USD 17.1) in routine outpatient clinics. This study is the first in Zambia to demonstrate the cost effectiveness of malaria diagnosis in the era of ACTs as treatment for uncomplicated malaria. In incremental analysis, the cost per additional case correctly diagnosed was found to be 70% (USD 7) lower for RDT than microscopy. It is more likely that policy makers would opt to implement RDTs given that they require less additional resources but also yield more correct diagnoses than microscopy, holding all other factors constant.

Microscopy malaria diagnosis in the peripheral health centres was less effective than expected (as compared by its assumed sensitivity). These findings challenge the notion that microscopy is the gold standard for malaria diagnosis [21,22]. This study proposes that microscopy is only gold standard when performed by expert microscopists under ideal conditions. The proportion of RDT false positives was found to be lowest among the three strategies (8%). These findings are within the range of the 10% false positives expected on HRP-2 based RDT [23]. It is therefore important to observe here that in terms of reducing false positives (and in turn reduce expenditure on unnecessary drugs) RDTs were more effective than microscopy and much more than clinical diagnosis.

The diagnostic test result did not seem to influence the decision to either treat or not treat with an antimalarial. It was found that almost 87% of all facility visits were prescribed antimalarials regardless of the malaria test result. However, an interesting observation was that the test result influenced the type of antimalarial, which was prescribed to a patient. Those cases that were more likely to have malaria (found positive) had a higher chance of being prescribed AL. Based on these observations; this study does not fully support the proposition that malaria diagnostic techniques do not guide treatment decision.

This study demonstrated that there are cost savings (although moderate) on treatment associated to a specific diagnostic test. This in spite of the treatment patterns discussed above. Moreover, assuming a situation where a diagnostic test result could strongly influence the decision to prescribe or not an antimalarial, microscopy and RDT diagnosis would have the potential of saving 56% and 59% respectively on antimalarials. In Malawi, it was found that using microscopy could lead to about USD14, 000 savings on drugs annually in one hospital [24]. In the Zambian context, it would be interesting to find out if the prescription trends found in the facilities (in this study) are similar in hospitals. This would provide an idea of the potential savings in antimalarials at that level of care.

Apart from the obvious effect on costs, irrational drug use may lead to stock outs of antimalarials at a time when they are most needed. Furthermore, the increase in drug pressure in the population could increase the probability of drug resistance to ACTs developing early [25,26]. The potential negative health effects this may have cannot be underestimated.

A study conducted by Rolland et al [18] in a hypothetical epidemic situation found the cost per true malaria case detected by RDT was USD 19.87 while for clinical diagnosis it was USD 18.4 at 25% prevalence. Rolland et al used a hypothetical epidemic situation and a different outcome measure hence making comparisons with this study difficult. However, in Thailand a study by Buolombai et al [27] compared the cost effectiveness of microscopy to two types of RDTs (OptiMAL and ICT) from a societal perspective. Microscopy was found to cost more per true *falciparum *positive case detected than the two RDTs (446.75 Baht vs 282.40 Baht and 343.56 Baht). Since this study is comparable in terms of using a longer data collection period and actual malaria setting (except for the costing perspective), the findings may be more comparable to the Zambian situation than the Rolland et al study. However, none of these studies compared all the three interventions as conducted in this study.

Thus when assessing the cost effectiveness of malaria diagnosis, differences in methodologies and patient population characteristics are very important and need to be explored more. Additionally, this study found that health workers might have a role to play in modifying the potential effectiveness of a test through their actual practice when diagnosing malaria. Hence future research should explore these areas and the extent to which they act as a confounding factor.

None of the parameters used in the sensitivity analysis changed the position of the RDT as the most cost effective strategy at correctly diagnosing malaria. However, some parameters increased the costs of cases correctly diagnosed, while others reduced the costs of case correctly diagnosed.

This study contributes to new knowledge on the economics aspects of malaria case management. This is relevant since economic evaluations on malaria interventions, especially in Zambia, are scarce. The results of this analysis will serve to inform policy makers on which alternatives will be most efficient in reducing malaria misdiagnosis by taking into account both the costs and effects of each strategy.

### Strengths

This study was conducted within the actual malaria context using field-based data in a malarious population. The advantage of field-based data is that it incorporates the inherent differences in practice, settings and seasons, which cannot be found in hypothetical or trial settings. The observation period was long enough to account for seasonal variations in malaria transmission which affects the expected patient visits and the prevalence of malaria at OPD. Variations in the study site settings provided an understanding into how facility utilisation, staffing levels, capital endowment and other capacities affect the cost of malaria diagnosis. The study also looked at all the possible outcomes of a diagnostic test as opposed to just focussing on the positive malaria cases. Sensitivity analysis on variables used in the estimations of costs and effects provided insights into how the study findings would change across other settings and contexts. This not only helped to show the robustness of the results but also showed how costs of malaria diagnosis may vary depending on the prevailing situation in an area being considered.

### Limitations

One of the main limitations of the study is that the outcome measure used in the analysis is an intermediate one. Thus it has been assumed that cases correctly diagnosed may be linked to improved final outcome (recovery from disease). However, the link between correctly diagnosing a case, an optimal clinical management of the patient and a satisfactory health outcome (after treatment) may be difficult to prove, without a close patient follow up. Thus within the realm of this study, it was not possible to estimate the link between incorrectly diagnosing a patient and the final clinical outcome (especially false negatives). This is a potential area for further research.

The use of a societal perspective is usually recommended in economic evaluations. However, in this study the provider perspective was used because malaria services are provided free of charge and the indirect patient costs would be similar across the four districts.

The use of facility registers may raise concerns with the reliability of the data due to potential errors in the data recording at facility level. Nonetheless, the use of a three level supervision system (facility, district and national level) during the entire 8 months ensured completeness and consistency in the data collection process.

Another limitation of the study arose from difficulties in defining the sensitivity of clinical diagnosis, as this can be very subjective. Further, clinical diagnosis does not allow for assessing the extent of to which patients should be suspected to have clinical malaria. However, other studies conducted elsewhere, have shown similar sensitivity values as the estimates found in this study [13,14].

## Conclusion

This study has shown that RDTs are the most cost effective method at correctly diagnosing malaria in lower level health facilities in Zambia when compared to clinical and microscopy strategies. However, the amount of cost savings in drugs was limited by the health worker treatment practices. The incremental cost per case correctly diagnosed and treated was USD 2.6 for RDT compared to USD 9.6 for microscopy. Thus RDT would be much cheaper to scale up than microscopy. The findings were robust to changes in assumptions and parameters. These findings could be relevant for transferability to low-income countries where malaria is endemic, AL is being used for first line treatment of malaria and microscopy diagnostic services are not readily available.

## Competing interests

The authors declare that they have no competing interests.

## Authors' contributions

PC participated in the design, implementation, data analysis and manuscript development. MCR and FM participated in design, data analysis and manuscript development.
